# Role of cortical excitatory/inhibitory imbalance in autism spectrum disorders from a symptom severity trajectories framework: a study protocol

**DOI:** 10.1186/s12888-023-04695-y

**Published:** 2023-03-29

**Authors:** Laura Colomar, Antonia San José Cáceres, Juan Álvarez-Linera, Javier González-Peñas, Abigail Huertas Patón, Daniel Martín de Blas, Ana Paloma Polo Arrondo, Andrea Solís, Emily Jones, Mara Parellada

**Affiliations:** 1 Instituto de Investigación Sanitaria Gregorio Marañón (IiSGM), Madrid, Spain; 2 School of Medicine, Universidad Complutense, Madrid, Spain; 3 CIBER de Salud Mental, Instituto de Salud Carlos III, Madrid, Spain; 4 Radiology Department, Hospital Ruber Internacional, Madrid, Spain; 5 Department of Child and Adolescent Psychiatry, Institute of Psychiatry and Mental Health, Hospital General Universitario Gregorio Marañón, Madrid, Spain; 6 Departamento de Bioingeniería, Universidad Carlos III de Madrid, Madrid, Spain; 7 Neurophysiology Department, Hospital General Universitario Gregorio Marañón, Madrid, Spain; 8 Centre for Brain and Cognitive Development, University of London, Birkbeck, London, UK

**Keywords:** Autism spectrum disorder, Excitatory/inhibitory imbalance, Trajectories, Symptom severity, Biomarker, Electroencephalography, Spectroscopy, Genetics

## Abstract

**Background:**

There is considerable evidence reporting an excitatory/inhibitory (E/I) cortical imbalance in autism spectrum disorders (ASD). However, previous findings on the direction of this imbalance and its relationship to ASD symptomatology are heterogeneous. Some factors contributing to these mixed results might be the methodological differences between studies assessing the E/I ratio and the intrinsic variability within the autistic spectrum. Studying the evolution of ASD symptoms and the factors that modulate it might help to explain and reduce this variability. Here we present a study protocol to explore the longitudinal role of E/I imbalance in ASD symptoms, combining different approaches to measure the E/I ratio and using the trajectories of symptom severity as a framework.

**Methods:**

This observational two time-point prospective study assesses the E/I ratio and the evolution of the behavioural symptoms in a sample of at least 98 participants with ASD. Participants are enrolled at 12 to 72 months of age and followed from 18 to 48 months after. A comprehensive battery of tests is applied to evaluate ASD clinical symptoms. The E/I ratio is approached from electrophysiology, magnetic resonance, and genetics. We will calculate the individual change for the main ASD symptoms and, based on that, we will define the trajectories of symptom severity. Then, we will investigate the correlation between measures of excitation/inhibition balance and autistic symptomatology cross-sectionally, as well as the ability of these measurements to predict changes in symptoms over time.

**Discussion:**

This study presents a robust multisystemic approach to the E/I imbalance theory in autism and its relation to divergent symptom trajectories. That setting will allow us to relate and compare the neurobiological information coming from different sources and its impact on behavioural symptoms while accounting for the high variability in ASD. The findings derived from this study could contribute to the ASD biomarkers research and might provide valuable evidence for the development of more personalized treatments in ASD.

## Background

While autism spectrum disorders (ASD) are defined within the group of neurodevelopmental disorders, their neurobiological bases remain poorly understood. The DSM-5 diagnosis of ASD is based on the presence of social-communication impairments, restricted, repetitive behaviours and interests, and sensory anomalies. This diagnosis can be further defined according to the individual’s cognitive abilities, language skills, the severity of the symptoms, and co-occurring conditions [1], leading to a very broad variety of phenotypical and biological profiles. Indeed, this heterogeneity is one of the factors hampering the identification of biomarkers for diagnosis and treatment.

It has been proposed that an imbalance between excitatory and inhibitory (E/I) cortical activity could underlie some forms of ASD [2, 3]. In their E/I imbalance model of ASD, Rubenstein and Merzenich [3] postulate that some forms of ASD might be caused by reduced activity of Gamma-aminobutyric acid (GABA), the principal inhibitory neurotransmitter in the neocortex. A reduction of GABAergic inhibition would impair the suppression of neural noise, leading to a lower signal-to-noise ratio, affecting neural processing [4, 5] and the opening of critical periods, ultimately increasing vulnerability for deviances from typical development [6, 7]. In the same vein, some other authors have proposed that some of the ASD behavioural symptoms might arise as an adaptive response to early widespread alterations in neural processing, such as an E/I imbalance [8]. From this point of view, the repetitive behaviours often observed in ASD could constitute an adaptative strategy to make the environmental information more predictable and thus, easier to process. Similarly, the lack of interest in social situations that some patients exhibit could represent an adaptive response of avoidance, given the high complexity and unpredictability of the social world [8, 9]. In line with this, some preclinical and shiftability studies have shown an improvement in autistic symptomatology with drugs that restore the inhibitory action of GABA [10–12].

Since the proposal of the E/I imbalance model of ASD [3], the putative role of the E/I imbalance in ASD has been approached using different techniques (for reviews see 13,14). To name some examples, Magnetic Resonance Spectroscopy (MRS) has been used to assess the E/I imbalance by estimating the cortical concentrations of GABA and Glutamate [13, 14]. Other studies have approached the E/I ratio measuring the blood plasma levels of GABA and glutamate [13]. Electroencephalography (EEG) and magnetoencephalography (MEG) have also been broadly used in E/I imbalance research. There is significant evidence from in vitro and in vivo studies showing the role of GABAergic interneurons in the generation of gamma band activity (30-100 Hz) in the brain [15, 16]. Similarly, beta oscillations (12-30 Hz) have been linked to GABAergic activity [17]. Based on that, the oscillatory activity in gamma and beta bands has been extensively employed as an indirect measure of the E/I ratio in ASD [13, 14, 18]. Beyond oscillatory proxies, the mismatch negativity (MMN) has also been widely studied in ASD. The MMN is a negative shift on the auditory event-related potentials (ERP) elicited when a deviant stimulus is presented. This shift typically takes place approximately 150-250ms post stimuli and lasts around 500ms [19]. MMN has been related to glutamatergic activity and specifically to N-methyl-D-aspartate (NMDA) receptors [20], making it a useful approach to the E/I ratio.

While there have been numerous studies in this field, the results obtained from the different techniques are heterogeneous and the existing literature in ASD shows considerable variability regarding the direction of this imbalance [13, 14]. EEG and MEG studies seem to show some consistency in finding reduced gamma-band activity in participants with ASD compared to controls in auditory tasks [14]. Also, studies using MRS suggest a reduction in GABAergic activity in peri-Ronaldic and temporal areas in individuals with ASD [14]. Genes regulating the E/I balance have also been associated with the ASD aetiology [21]. Conversely, electrophysiological studies using other tasks (such as resting-state or visual tasks) and MRS studies focused on glutamate (as the main excitatory neurotransmitter in the neocortex) report mixed findings [14]. Similarly, heterogeneous findings have been reported using other techniques, such as transcranial magnetic stimulation, or blood measurements of Glutamate and GABA levels [13, 14]. In summary, existing research suggests that E/I imbalance might play a critical role in ASD. However, there is still uncertainty about the specifics of this imbalance and its impact on critical windows for the expression of the clinical phenotype of ASD.

Many factors can contribute to the inconsistencies in the literature, including differences in the techniques employed, study settings, data processing, and data analysis. In this scenario, combining different approaches to measure the E/I ratio in the same sample could provide valuable insight into the consistency and reliability of the E/I measurements. Another aspect that might add to the incongruences among studies is the intrinsic heterogeneity of ASD [22]. The impact of E/I alterations in the autistic phenotype might vary across different forms of ASD. Furthermore, it could be modulated by a series of external factors, such as age or sex. If we assume that the ASD symptoms arise as an adaptive response to early E/I alterations, then studying the relationship between the evolution of the symptoms and the changes in the E/I ratio across development would be key. In this context, the study of trajectories of symptom severity would allow us to define the evolution of the symptoms based on the magnitude and direction of their changes and assess the effect of other variables on symptom evolution. Existing studies of trajectories of symptom severity in ASD show some variation in their findings. While some authors have reported that most of the participants (over 80% of the sample) follow a stable trajectory [23, 24], some other studies reported that to be the case only for the 50% of the sample approximately [25, 26]. When exploring the factors related to change, improving trajectories have been related to high socioeconomic status [27], greater cognitive and language skills [28] and sex, with significantly more females improving in their symptoms [24–26]. Some studies reported that improving trajectories were also related to higher initial intelligence [23, 25] but some others failed to find this effect [26].

The study of the covariation of the E/I ratio and the ASD symptoms trajectories, while controlling for other factors related to symptoms change, might be key for understanding the role that E/I alterations might have on the ASD phenotype and its downstream effects on development. The main objective of this project is to study and characterize the role of E/I cortical imbalance in the evolution of ASD behavioural symptoms. To do that, we aim (I) to examine the trajectories of ASD symptom severity in a cohort of children with ASD, (II) to approach the E/I ratio combining three techniques: EEG, MRS and genetics, and (III) to investigate the relationship between the E/I ratio and ASD symptoms longitudinally, using the trajectories of symptom severity as a framework. Finally, participants whose symptoms evolved in a similar way might be more likely to present common neural alterations. Thus, we would like to explore if distinct trajectories of symptom severity might be associated with different alterations in the E/I ratio.

## Methods

### Study overview and setting

The study here presented is an ongoing observational prospective cohort study taking place at the Department of Child and Adolescent Psychiatry of Hospital General Universitario Gregorio Marañón (Madrid, Spain). In this study, we aim to explore the role of E/I cortical imbalance over ASD symptoms longitudinally. Relevant clinical and behavioural information is collected through a comprehensive battery of tests and evaluations at two points, 18 to 48 months apart, and the E/I imbalance approached using EEG, Magnetic Resonance (MR) and genetics profiling.

Completing the clinical assessment is necessary to be included in the study, but participation in the EEG, MR, and genetic testing is optional and depends on the participants’ ability to withstand these assessments. Once the parents agree for their child to participate, the study schedule is arranged depending on their preferences. Typically, participants take a blood test and complete the clinical evaluations and the EEG session during a day-long visit, with a lunch break between assessments. Then, the MR study is scheduled for a subsequent visit. While proximity among appointments for the administration of the different techniques is preferred, a separation of up to 6 months between assessment points is allowed. In the second data collection point, 18 to 48 months later, participants are encouraged to repeat all the evaluations they completed on their first visit, except for the genetic test which should only be taken once.

### Ethics

The study was approved by the Ethics Committee for Research with Medicines of Hospital General Universitario Gregorio Marañón (reference number: CLU-02) and conducted in accordance with the Declaration of Helsinki. Due to the legal minority of all participants, written informed consent is obtained from the participants’ parents or legal guardians before inclusion in the study.

### Recruitment

Participants are recruited from the ASD Complex Diagnosis Service of the Hospital General Universitario Gregorio Marañón (Madrid, Spain). This service is provided by a multidisciplinary team including nurses, psychiatrists, psychologists, and social workers. It evaluates children and adolescents suspected of ASD. After diagnosis, if a participant fulfils the inclusion criteria, their parents are briefly informed about the study by their psychiatrist shortly after they receive the diagnosis. If they are interested in the study, a researcher provides further details and sets the appointments.

The inclusion criteria for participation in the study are: (I) having a diagnosis of ASD or Pervasive Developmental Disorder-Not Otherwise Specified (PDD-NOS) according to DSM-5 or DSM-IV criteria and (II) being aged between 12 and 72 months old at enrolment. Any patient with a severe or unstable condition, is excluded from the study.

### Measurements

#### Assessment of ASD clinical symptoms

One of the main objectives of this study is to identify trajectories of ASD symptom severity. To that end, we evaluate core ASD symptoms at two time points. We complete this evaluation with further relevant clinical and sociodemographic information that might have an impact on the evolution of the symptoms.

For the assessment of core ASD symptoms, we combine different tests and questionnaires. Current ASD symptoms are measured with the *Autism Diagnostic Observation Schedule, Second Edition (ADOS-2)* [29]. The ADOS-2 is a standardized, semi-structured diagnostic tool based on the direct observation of behaviours in the areas of communication, social interaction, play, sensory processing, and restricted and repetitive behaviours. In our study, the ADOS-2 is applied by a psychologist with ADOS-2 reliability, and the application is videotaped for posterior consensus scoring, if necessary. Due to COVID-19’s safety measures, some evaluations in some settings (i.e., health establishments) require the use of face masks by both the child and the examiner making the coding of some items (e.g., B2) not possible. The total number of children affected by these measures will be reported and taken into consideration for further analyses. We also score the Childhood Autism Rating Scale, Second Edition (CARS2) [30], based on the ADOS-2 administration and on the *CARS2 Questionnaire for Parents or Caregivers.* The CARS2 is a standardized instrument that informs about symptom severity in ASD, based on the frequency, intensity, particularity, and duration of autistic behaviours. The *Social Responsiveness Scale, Second Edition (SRS-2)*[31] is used as a parent-report measure of ASD symptoms over the previous 6 months. The SRS is composed by two sub-scales that provide information about the symptoms in the social communication and interaction (SCI sub-scale) domain and about the restricted interests and repetitive behaviours (RRB sub-scale). Finally, the *Repetitive Behaviour Scale-Revised (RBS-R)* [32] and the *Sensory Profile 2, Caregiver form* [33] are used as parents-reported measures of restrictive and repetitive behaviours and sensory processing, respectively.

For assessing intelligence, we use the *Weschler Preschool and Primary Scale of Intelligence-IV* (WPPSI-IV)[34], which is a suitable scale for measuring intelligence in children ranging from 2 years and 6 months to 7 years and 7 months old. Adaptive behaviour for daily life activities, is evaluated using the *Adaptive Behaviour Assessment System, Second Edition (ABAS-II)* questionnaire [35] informed by the parents.

Socioeconomic and demographic information is collected with the participants’ parents or legal guardians using a locally devised interview. This interview also contains information about the onset of the symptoms and developmental milestones and further clinical variables that might be relevant when studying the evolution of the symptoms such as diet, and concomitant illnesses. For information about the pregnancy and birth, we use the *Murray-Lewis Obstetric Complications Scale* [36].

Participants are recruited shortly after they receive their diagnosis. Therefore, frequently they have recent administrations of the ADOS-2, CARS2 and/or WPPSI-IV at enrolment. If that is the case, these evaluations scores are used in the study to avoid repeating the assessments.

#### Electroencephalography

The EEG session consists of a set of four visual and auditory passive tasks, with a total duration of 30 min approximately. During the EEG session, participants are asked to sit calmly in an armchair placed approximately 90 cm away from the screen, with two speakers situated at either side of the screen. To facilitate participants’ collaboration, one parent is allowed to be present during the EEG assessment. Parents are asked to remain quiet and not to interact with their children during the stimuli presentation. To avoid excessive movement, participants can sit on their parent’s lap.

Electrophysiological activity is collected through a 20-channel helmet, and it is recorded using the ENOBIO 20 System and the NIC2 Software (Neuroelectrics ®). The visual and auditive stimuli are presented in the participant’s screen using the MATLAB open toolboxes Psychtoolbox [37] and Psychport Audio. The following tasks are presented:

- A resting-state with a social and a non-social condition. In the social condition, participants are presented a video with two women singing nursery rhythms. In the non-social condition, some toys in action are presented. Each video lasts one minute, and they are presented twice during the session.
- A reversal checkerboard task. The stimuli consist of black and white pattern reversal checkerboards of 32 × 32 checks with a red cross fixation at the centre of the display. A reversal of the checkerboard pattern occurs every 500ms. Four blocks of 90 stimuli are presented, and each block lasts 45s. Blocks are counterbalanced, so two of them start with the black checkerboard and the other two start with the white checkerboard.
- A pure tones task at frequencies of 200, 300, 500, and 1000 Hz of 300ms duration each (including 10ms ramps). The interstimulus interval is 1000ms ± 100ms, jittered. We present one hundred thirty tones at each of the four frequencies, randomly. The total duration of the task is about 11 min. While listening to the tones, participants can watch a silent movie.
- An auditory oddball task. Here, we present two pure tones: tone A (1000 Hz tone, 50ms duration) and tone B (1000 Hz tone, 100ms duration). The intertrial stimuli are 400ms ± 100ms, jittered. The standard stimulus is presented 85% of the time and the deviant stimulus is presented 15% of the time. Stimuli are presented in two counterbalanced blocks, so each tone is the standard tone in one block and the deviant tone in the other block. In total, 740 tones are presented in each block. The total duration of this task is approximately 12 min. During this task, participants are presented a silent movie.

The tasks are always presented in the same order. There is a manually controlled pause after every task or block, so that the duration of the pause can be adapted to the participant’s needs.

#### Magnetic resonance

We use MRS to measure the concentrations of cortical glutamate and GABA, the main excitatory and inhibitory neurotransmitters in the neocortex. Besides MRS, participants undergo structural magnetic resonance imaging (MRI), high angular resolution diffusion-weighted imaging (HARDI) and resting-state functional magnetic resonance imaging (rs-fMRI). Structural scans are acquired for clinical purposes, MRS voxel placement, and to provide complementary anatomical information. Every participant’s family are given a clinical report of the structural MRI. HARDI data is acquired to study white-matter microstructure and to estimate structural connectivity. Finally, rs-fMRI data provide both local and long-range functional connectivity metrics.

Scans are acquired using a 3T Magnetom Prisma Siemens (Erlangen, Germany). We use a 32 channels head coil and a system of head immobilization to guarantee the quality of the acquisitions. The MR session has a duration of approximately 45 min in which the participants are sedated. Sedation was determined necessary given the participants age and symptomatology.

The sedation protocol for the MRI assessment, which has been used in previous neuroimaging studies [38], consist in nasal anesthesia administration, by an anaesthesiologist, while monitoring the patients. Propofol is administered intravenously and kept as the minimum possible dose to maintain the patient motionless while keeping the child’s airway unobstructed with a larynges mascara. The duration of sedation is short, allowing fast recovery times.

Sequences are, in order of acquisition, as follow:

- A high-resolution T1-weighted structural image is acquired using a three-dimensional (3D) MPRAGE sequence with the following parameters: field of view (FOV) = 224 × 224 × 160mm3; voxel size = 1 × 1 × 1mm3; time of echo (TE) = 2.25ms; time of repetition (TR) = 2300ms; inversion recovery time (TI) = 900ms; flip angle = 9°; Acceleration factor GRAPPA = 2, acquisition time (TA) = 312 s (5:12 min).
- Single-voxel proton MRS data is acquired in two different regions of interest (ROI): medial parieto-occipital cortex (mPOC) and anterior cingulate cortex (ACC). Volume of interest (VOI) are 30 × 30 × 30mm3 for the mPOC and 30 × 37 × 24mm3 for the ACC. For both ROIs a MEGA-PRESS sequence with weak water suppression and the following parameters is employed: TR = 2000 ms; TE = 68 ms; 96 averages; bandwidth = 1200 Hz; ON-editing frequency = 1.9ppm; OFF-editing frequency = 7.5ppm; editing bandwidth = 50 Hz; TA = 392s (6:32 min). Additionally, for each ROI, an auxiliary MEGA-PRESS sequence with the same parameter but without water suppression and only 10 averages is acquired (TA = 48 s).
- HARDI images are acquired using a multi-shell diffusion-weighted scheme with two shells of b-values of 1000s/mm2 and 2000s/mm2, with 32 and 64 directions, respectively. Additionally, 6 non-diffusion-weighted (i.e. with b = 0s/mm2) volumes are included in the sequence in an interleaved way. A spin-echo single-shot echo-planar imaging (EPI) sequence with the following parameters was used: 66 axial slices, 2 mm slice thickness no gap; 2 mm isotropic voxels; phase-encoding (PE) direction = Anterior-to-Posterior; TE = 68 ms; TR = 4000ms; flip angle = 9º; FOV = 220 mm; Acceleration factor GRAPPA = 2 x SMS (Multiband) = 2; TA = 444s (7:24 min). An auxiliary sequence of 6 non-diffusion-weighted volumes is also obtained with the same parameters but opposite PE direction (TA = 50s).
- Rs-fMRI images are composed by 250 volumes acquired using a gradient-echo single-shot EPI sequence with the following parameters: 54 contiguous axial slices, 2.5 mm thickness; 2.5 mm isotropic voxel; PE direction = Anterior-to-Posterior; TR = 1500ms; TE = 30ms; flip angle = 70º, FOV = 220 mm; Acceleration factor SMS(Multiband) = 3; TA = 385s (6:25 min). Two auxiliary images, with 4 volumes each, are acquired using a spin-echo single-shot EPI sequence, both with the same geometry and readout as the rs-fMRI images, but one with same PE direction and the other with opposite PE direction. Additional parameters for this sequence are: TE = 48ms; TR = 4510ms; flip angle = 90º; TA = 23s.

#### Genetics

This study also includes a genetic test using DNA from blood samples. The blood sample is drawn by nurses who are familiarized with the care of patients with ASD. Blood samples are stored at -80ºC for later analyses.

### Data collection and management

Different professionals contribute to the data collection at different stages of the study. The blood sample is drawn by nurses. The clinical evaluation with the participants and the interview with their parents are conducted by a psychiatrist or a psychologist with experience working with children with ASD. The EEG and MR sessions are conducted by two neurophysiologists, an anaesthesiologist and a neuroradiologist, respectively.

Once the information is collected, the questionnaires and case report forms are stored physically in the hospital, pseudoanonymized. The association between the study identifiers and participants’ contact data is stored in a password-secured database, with exclusive access for the principal investigator (PI) and the study coordinators. All the information is stored and managed using Research Electronic Data Capture (REDCap) hosted at Instituto de Investigación Sanitaria Gregorio Marañón. REDCap is web application for the secure online storage and management of databases [39, 40]. Only the PI, the data entry operator, and the study coordinators have access to the database. The videorecording of the ADOS-2, and the EEG and MR files are stored in an encrypted server under hospital regulations. Access to any of the study data for research purposes needs to be approved by the PI.

### Statistical analysis

#### Identifying trajectories of symptom severity

For the calculation of trajectories of symptom severity, we will focus in three areas: socio-communicative symptoms, restricted interests and repetitive behaviours, and sensory alterations. We will calculate the trajectories of socio-communicative symptoms based on the ADOS-2 Calibrated Severity Scores for the Socio-Affective sub-scale (ADOS-2 SA CSS) and on the SRS-2 SCI raw scores. The ADOS-2 scores are a measure of current symptomatology based on clinical observation, while the SRS-2 provides information about the symptoms over the last six months reported by parents. Similarly, for the restricted interests and repetitive behaviours, we will use the ADOS-2 Calibrated Severity Scores for the Repetitive and Restricted Behaviours sub-scale (ADOS-2 RRB CSS), the SRS-2 RRB raw scores, and the RBS-R total scores. Finally, we will use the Sensory Profile-2 scores to define trajectories of sensory symptom severity.

For these measures, we will calculate individual change per year for each participant. Individual change will be defined as the difference between the scores in the two data collection points, divided by the time between visits. To determine if the individual change is significant, we will stablish a threshold considering the variance among participants and the variance within participants across time. Based on that threshold and the individual change scores, we will classify the participants in three groups: significantly improving trajectory, stable trajectory, and significantly worsening trajectory.

For a greater characterization of the trajectories, we will use ANOVA (or Kruskal-Wallis tests, for non-normally distributed data) for testing for differences between the three trajectories in intelligence, adaptive behaviour, age at enrolment, socio-demographic variables, and other relevant clinical information such as perinatal variables, diet and concomitant illnesses. We will apply Bonferroni correction to control for multiple testing. Then, we will use multiple regression to evaluate the effect that the named variables might have on the change of the main symptoms.

#### Assessing E/I imbalance

We will study the E/I ratio using different proxies depending on the technique and task used. For the EEG, we will base our analyses in mainly two markers: the gamma-band activity and the MMN. We will analyse gamma activity in the data coming from the pure tones task, the checkerboards task, and the resting state. We will study the MMN in the auditory oddball task data. Finally, while these are the primary EEG markers we will rely on, some more novel E/I markers might be considered [18]. Specifically, the aperiodic signal of the power spectral density slope, known as the 1/f component, has shown to reflect changes in E/I balance [41] and might allow us to compare the results across the different tasks and modalities.

For the MRS, analysis of acquired MEGA-PRESS difference spectra will be conducted with LCModel [42] to extract concentrations of GABA and glutamate in our two ROIs (mPOC and ACC). Moreover, the MEGA-PRESS OFF spectral will also be analysed to obtain additional estimations of glutamate concentrations. For the fitting of the spectra, we will use a metabolite basis simulated with FID-A [43] using the parameter of the MEGA-PRESS sequence implemented. For both MRS ROIs, using the structural MRI data, we will quantify the amount of grey-matter, white-matter and cerebrospinal fluid present in the VOI. Furthermore, as secondary and exploratory measures, abnormal white matter microstructure, as well as structural and functional connectivity, will be studied through HARDI and rs-fMRI, and their correlations with clinical trajectories analysed. HARDI data will be processed using FSL [44], MRTrix3 [45] and SMT [46] toolboxes obtaining voxel-wise maps of white-matter microstructure metrics (as diffusion-tensor fractional anisotropy or intraneurite volume fraction), atlas-based structural ansiostropy or intraneurite volume fraction, and atlas-based structural connectivity matrices. Rs-fMRI data will be processed using FSL and the python package *nilearn* to obtain regional homogeneity maps and both voxel-wise and atlas-based functional connectivity matrices.

Regarding genetics, we will focus on rare coding genetic variation that has established association with ASD, such as de novo protein-truncating and missense mutations, as well as rare inherited variants that also disrupt genes.

#### Relationship between E/I imbalance and behavioural symptoms

First, we will explore the cross-sectional relationship between the E/I ratio and ASD symptoms in the first data collection point using correlations and structural equation models. Then, we will study the relationship between the E/I ratio and ASD symptoms over time. To that end, we will calculate change for the different measurements of the E/I imbalance defined as the difference between time points one and two, divided by the time between visits. We will study the longitudinal relationship between the E/I ratio and ASD symptoms using two approaches. For the first approach, we will consider the change in ASD symptoms as a continuous variable and we will calculate its correlation to the E/I ratio at the first time point and the change in the E/I ratio across time. Then, we will use structural equation models to study the putative role that the E/I ratio might have over the change in ASD symptoms. In the models, we will also include clinical and demographical variables that might have an impact on change. For the second approach, we will consider the change in ASD symptoms as a categorical variable distinguishing three groups: improving, stable and worsening trajectories. Then, we will test if there are differences between the three groups in the E/I ratio at the first time point and in the change in the E/I ratio across time. To do this, we will use ANOVA or Kruskal-Wallis tests, depending on the distribution of the variables. To conduct these analyses, we will use Principal Component Analysis (PCA) to lower the dimensionality of E/I data. This will reduce the complexity of the data and might improve the interpretability of the results.

#### Sample size

We will study the relationship between E/I imbalance and the evolution of ASD symptoms from a continuous perspective (based on the individual change scores) and from a categorical perspective (based on the three trajectories classification: improving, stable and worsening). Thus far, data studying the relationship between E/I imbalance and change in severity of ASD symptoms are very limited. Therefore, sample size calculations were based on studies of the cross-sectional correlation between E/I imbalance and ASD symptoms. Previous EEG/MEG studies exploring the relationship between E/I imbalance and ASD symptoms reported correlation coefficients slightly above 0.30 [47–49]. Based on that, we estimated a correlation coefficient between E/I proxies and change in ASD symptoms of 0.30. Thus, a sample size of at least 85 participants was stablished assuming an alpha of 0.05 and a beta of 0.2 (meaning a power of 80%). We expect the attrition rate to be a 15%, therefore the estimated minimum sample size would be 98 participants.

## Discussion

This study aims to explore the role of E/I imbalance in ASD symptoms longitudinally. To date most of the studies assessing the role of E/I imbalance in ASD have relied on a single technique (i.e.: MRS, EEG) and, frequently, on one data point. These facts have contributed to the heterogeneity of findings and the lack of consensus in the field. To overcome some of the limitations of previous studies, in this project we propose to assess E/I ratio using three techniques, and two data collection points, using trajectories of symptom severity as a framework.

### Markers of E/I ratio

For approaching E/I ratio, we selected a set of techniques and tasks, based on previous evidence that would fit the characteristics of our sample (i.e., young children with or without intellectual disability). For the EEG assessment, we designed a battery with two auditory, one visual, and a resting state task. Auditory tasks have been one of the main focuses in EEG/MEG studies approaching E/I imbalance in ASD, and previous studies have reported differences in auditory gamma-band activity between participants with ASD and controls [14]. The MMN response elicited during auditory oddball tasks has also been of interest in E/I imbalance research. MMN has been found to be reduced in ASD, with participants with ASD exhibiting lower amplitude and/or greater latencies than controls [50]. As a visual task, we selected a checkerboards task. Given the high complexity of visual processing, the integration and interpretation of results in visual tasks is generally harder than in auditory tasks and can be further affected by participants attention. Thus, we prioritized a simple stimuli and short task that could suit young, poorly collaborative participants. Finally, we included an EEG resting-state task. Resting-state gamma-band activity has been reported to be reduced in children with ASD compared to controls and related to SRS scores [49].

Regarding the MR assessment, MRS has shown to be a useful technique for assessing E/I imbalance in ASD. Indeed, previous studies have reported reduced levels of GABA and increased levels of glutamate in the ACC [51–53] and occipital cortex [54] in patients with ASD compared to controls. Rs-fMRI and HARDI allow the study of white matter microstructure, as well as functional and structural brain connectivity. Previous literature reported a share pattern of abnormal structural and functional connectivity in ASD compared to neurotypical population (for a review see [55]).

Finally, by performing whole-exome sequencing in large cohorts, recent studies have reached enough power to report genes significantly associated to the genetic aetiology of ASD, with many of them modulating the excitatory and inhibitory neural functioning [21]. In this sense, a recent study on human brain cells during prenatal stages has also reported enrichment in excitatory and inhibitory neurons in ASD [56]. In our study we will focus on those rare coding genetic variation that have been associated with ASD and E/I imbalance.

We believe that the combination of different E/I ratio proxies will provide valuable information about the nature of the biomarker *per se* and how it expresses across the different tasks and techniques. Thus, this study might shed some light on the integration and interpretation of previous evidence coming from different fields. Furthermore, it could constitute preliminary evidence regarding the differential information that each technique can provide, and which would be the best choice in different situations (i.e., which parameter shows more variability across time, which one shows greater relationship to ASD symptoms, etc.).

### Limitations

There are some limitations that we could face during the development of the project. First, due to the young age of the participants, compliance during some assessments such as the EEG or the blood test may be hard. To minimize this from happening, we have put measures in place such as sharing with them videos about the different procedures in the study, allowing parents to be present at all times or incorporating user-controlled pauses between the EEG tasks. Second, the study’s enrolment age (12 to 72 months) may encompass significant variability among children. This range was deemed necessary to guarantee the inclusion of a representative sample of recently diagnosed children. To account for this variability, the age at enrolment will be considered as a covariate in the statistical analysis. Furthermore, as is usual in longitudinal studies, we might face some sample loss in the follow-up visit. Thus, we increased the sample size by 15% to deal with attrition. Regarding the MR study, the usage of sedation (Propofol) during the acquisition may affect the MRS metabolite quantification and rs-fMRI derived metrics. Nevertheless, minimizing the participants movements during the MR acquisition is crucial for obtaining usable data and other strategies used with ASD children, as acquiring while the participant is sleeping, will also affect the functional connectivity metrics. To control for this, the Propofol dose administered during sedation will be included as covariate in the statistical analysis. Finally, the scarce evidence so far with regards to the neurological basis of autism, particularly of different subtypes of autism, hinder establishing more specific hypothesis.

### Conclusions and clinical implications

The study of the longitudinal relationship between the E/I ratio and ASD using trajectories of symptom severity might help to disentangle the role that E/I alterations might have in the development of the autistic phenotype, while accounting for the high variability within ASD. Furthermore, it could inform about the potential neurobiological differences that might underlie different ASD phenotypes, improving the research of more personalized treatments. Finally, the combination of different techniques and tasks for approaching the E/I ratio might provide a better understanding of its nature and of the congruency and comparability within measures, contributing to the research on ASD biomarkers and its translation to the clinical practice.

## Data Availability

The data generated during the current study will not be publicly available since it contains clinical information that could compromise the participants’ privacy, but it will be available from the corresponding author on reasonable request.

## Abbreviations

ABAS-II: Adaptive Behaviour Assessment System, Second Edition
ACC: Anterior Cingulate Cortex
ADOS-2: Autism Diagnostic Observation Schedule, Second Edition
ADOS-2 CSS: ADOS-2 Calibrated Severity Scores
ADOS-2 RRB: ADOS-2 Repetitive and Restricted Behaviours sub-scale
ADOS-2 SA: ADOS-2 Socio-Affective sub-scale
ASD: Autism Spectrum Disorder
CARS2: Childhood Autism Rating Scale, Second Edition
DSM-IV: Diagnostic and Statistical Manual of Mental Disorders, Fourth Edition
DSM-5: Diagnostic and Statistical Manual of Mental Disorders, Fifth Edition
EEG: Electroencephalography
E/I: Excitatory/Inhibitory
EPI: Echo-Planar Imaging
ERP: Event-related potentials
FOV: Field of view
HARDI: High Angular Resolution Diffusion Imaging
GABA: Gamma-aminobutyric acid
IQ: Intelligence Quotient
MEG: Magnetoencephalography
MMN: Mismatch Negativity
mPOC: medial Parieto-Occipital Cortex
MR: Magnetic Resonance
MRI: Magnetic Resonance Imaging
MRS: Magnetic Resonance Spectroscopy
NMDA: N-methyl-D-aspartate
PCA: Principal Component Analysis
PDD-NOS: Pervasive Developmental Disorder-Not Otherwise Specified
PE: Phase Encoding
PI: Principal Investigator
RBS-R: Repetitive Behaviors Scale-Revised
REDCap: Research Electronic Data Capture
ROI: Region of interest
rs-fMRI: Resting-state Functional Magnetic Resonance Imaging
SRS-2: Social Responsiveness Scale, Second Edition
SRS-2 RRB: SRS-2 Restricted Interests and Repetitive Behaviour sub-scale
SRS-2 SCI: SRS-2 Social Communication and Interaction sub-scale
TA: Acquisition Time
TE: Time of Echo
TI: Inversion Recovery Time
TR: Time of Repetition
VOI: Volume of Interest
WPPSI-IV: Weschler Preschool and Primary Scale of Intelligence-IV
